# Yes-associated protein-based detection of acute type A aortic dissection and evaluation of therapeutic responses

**DOI:** 10.3389/fcvm.2024.1432007

**Published:** 2024-12-23

**Authors:** Kai Zhu, Hao-Xi Li, Dong-Dong Wu, Si-Chong Qian, Xiao-Long Wang, Jian-Rong Li, Wen-Jian Jiang, Hong Liu, Hai-Yang Li, Hong-Jia Zhang

**Affiliations:** ^1^ Department of Cardiovascular Surgery, Beijing Anzhen Hospital, Capital Medical University, Beijing, China; ^2^ Beijing Lab for Cardiovascular Precision Medicine, Beijing, China; ^3^ School of Public Health, Capital Medical University, Beijing, China; ^4^ Department of Cardiac Surgery, Fujian Medical University Union Hospital, Fuzhou, China; ^5^ Department of Cardiovascular Surgery, The First Affiliated Hospital of Nanjing Medical University, Nanjing, China

**Keywords:** acute aortic dissection (AAD), acute coronary syndrome (ACS), yes-associated protein (YAP), early diagnosis, major adverse events (MAEs)

## Abstract

**Background:**

Acute aortic dissection is a lethal cardiovascular emergency; early diagnosis is critically necessary. Novel serum biomarkers can potentially help in early detection and estimation of postoperative outcomes. Yes-associated protein (YAP) is a critical effector of the Hippo pathway, our aim was to explore the association between YAP and the diagnosis and prognosis of AD.

**Methods:**

We prospectively recruited 110 consecutive chest-pain patients [acute type A aortic dissection (ATAAD), *n* = 60; acute coronary syndrome (ACS), *n* = 50]. Blood samples were collected to determine levels of YAP and other serum biomarkers, and receiver operating characteristic curves (ROC) were constructed to assess the predictability in early diagnosis of AAD and postoperative major adverse events (MAEs).

**Results:**

YAP concentration was substantially elevated among ATAAD patients [3.45 (3.18, 3.63) vs. 2.44 (2.23, 2.59), *P* < 0.01]. Moreover, the white blood cell (WBC) count and plasma fibrin D-dimers were remarkably high among ATAAD patients [11.46 (9.57, 14.03) vs. 6.24 (5.10, 7.30), *P* < 0.01; 2,097.00 (998.75, 3,652.00) vs. 97.00 (67.25, 137.00), *P* < 0.01]. The serum YAP level held as a good predictive value in early diagnosis of ATAAD. The optimal cutoff value was 3.15 ng/ml, with an AUC of 0.94 (95% CI, 0.90–0.98, *P* < 0.01), sensitivity of 80% and specificity of 98%. The combined model of YAP, WBC count and D-Dimer exhibited an enhanced predictive power, with an AUC of 0.99 (95% CI, 0.98–1.00, *P* < 0.01). Serum YAP values at 12 h post-surgery provided the most accurate prediction of postoperative MAEs, with an optimal cutoff value of 3.60 ng/ml, an AUC of 0.89 (95% CI, 0.79–0.99, *P* < 0.01), and sensitivity and specificity of 88% and 82%, respectively.

**Conclusions:**

The serum YAP concentration is an early and sensitive serum bioindicator for distinguishing AAD from ACS. Moreover, the amount of postoperative circulating YAP is a robust indicator of postoperative MAEs.

## Introduction

Acute aortic dissection (AAD) is an often-lethal consequence of cardiovascular disease. This condition deteriorates quickly, and has an early untreated mortality rate of approximately 1%–2% per hour once symptoms arise (1). Given its critical and highly fatal nature, it is imperative to make well-defined and standardized diagnosis and treatment as soon as possible. Among the primary challenges of a definitive AAD diagnosis is the delineation of this condition from other sudden-onset severe chest pain disorders, particularly, acute coronary syndrome (ACS) and acute pulmonary embolism (APE), which often present with similar symptoms, but require different interventions. As ACS has always been the first diagnosis considered in patients with chest pain. AAD misdiagnosis as ACS often worsens AAD symptoms, and results in rupture or patient death disorders, particularly, when thrombolytic drugs even percutaneous endovascular angiography are employed inappropriately (2, 3). Moreover, in these cases, typical diagnostic tools, such as, electrocardiogram (ECG), computerized tomography (CT) and myocardial troponin lacked diagnostic sensitivity and specificity (4). Aortic computed tomography angiography (CTA), as the preferred diagnostic technique, may be limited or not available in the emergency room, especially in lower-level city and country hospitals. It is reported that the complexity of the clinical symptoms of AAD has a high probability of misdiagnosis, with up to 40% of cases being confirmatory diagnosed only after postmortem (5, 6).

Peripheral blood biomarker detection is typically a simple and mature procedure, with relative stability, short duration and reduced cost. There is a critical clinical need for novel potent bioindicators that can aid in the early detection of AAD. Currently, D-dimer is the only widely-recognized diagnostic biomarker of suspected AAD (7–9), and its detection method is reliant on the over-activation of the fibrinolytic system (10). Unfortunately, augmented D-dimer content is also prevalent among other thrombus or clotting diseases such as APE (11). Therefore, it is often challenging to delineate AAD from other such disorders using D-dimer. Hence, it is often used as an exclusion marker, with certain limitations. Moreover, among patients with false lumen thrombosis or reduced extensive disease, the values may have decreased specificities (12). Of course, a biomarker that provides additional information regarding the reliable inclusion or exclusion of AAD diagnosis is extremely valuable, particularly, if assessment can be made at symptom onset.

Yes-associated protein (YAP) is a critical effector of the Hippo pathway. It is the best-known sensor of microenvironmental mechanics, is regulated by extracellular matrix stiffness (13), and widely involved in the regulation of cell proliferation and apoptosis (14, 15). The critical role of YAP has also been confirmed in cardiac vascular smooth muscle cells (VSMCs) proliferation during cardiovascular development (16).

Multiple investigations, till date, explored the association between YAP and AD pathogenesis. Our previous study identified reduced expression of YAP in human acute type A aortic dissection (ATAAD) samples (17). In addition, smooth muscle-specific YAP-KO mice exhibit spontaneously developed aneurysms brought on by elastin disarray, SMC apoptosis, proteoglycans accumulation and aberrant immune cell populations (18). More importantly, alterations in YAP expression during AAD pathogenesis is remarkably distinct from other mechanisms that cause chest pain, namely, coronary artery syndrome and pulmonary embolism (19, 20). Relative to traditional biomarkers, YAP expression may, therefore, be able to delineate AAD from other acute chest pain syndromes. Furthermore, altered YAP expression is reported to correlate with mechanical stress disruption and extracellular matrix (ECM) damage among ATAAD patients (17). Herein, we explored whether YAP is a promising candidate as an early sensitive AAD biomarker, and whether it predicts postoperative efficacy of AAD patients.

## Methods

### Study design

We have obtained ethical approval from Beijing Anzhen Hospital, Capital Medical University (approval number: 2018004). Since all procedures were within the normal routine intensive care routine with no additional risk to patients, and the blood used for testing was a by-product of routine care, our institution's Ethics Committee granted the informed consent exemption. All human-related protocols strictly followed the revised guidelines of the Declaration of Helsinki (2013). Data collection was performed prospectively.

### Patients

In all, 110 consecutive chest-pain patients (ATAAD, *n* = 60; ACS, *n* = 50) were recruited from our Department of Cardiovascular surgery between July 2022 and November 2022. The following patients were selected for analysis: aged between 18 and 80 years; with ATAAD or ACS diagnosis definitively; cardiovascular surgery was performed. The following patients were eliminated from: severe neurological injury with complications; myocardial or internal organ malperfusion producing severe dysfunction. Upon admission, blood samples were collected for YAP evaluation at five distinct time points: at admission (T0), 12 h post operation (T1), 24 h post operation (T2), 48 h post operation (T3), and 72 h post operation (T4). Blood samples were collected by two experienced doctoral medical students, and they were blinded to patient intervention. Additionally, clinicians were blinded to patient YAP concentration data. Subsequently, patient management followed normal treatment procedures of cardiac surgery.

The ATAAD patients were separated into two categories, depending on the occurrence of major adverse events (MAEs) following surgery. MAEs were considered if any of the four following conditions were present: permanent neurological dysfunction (PND), paraplegia, new postoperative acute kidney injury (AKI) requiring continuous renal replacement therapy (CRRT), and mortality. A localized neurological deficit was designated as PND, with further confirmation as a new deficiency using brain CT scanning or magnetic resonance imaging. Individual organ malperfusion was defined as impaired blood flow to corresponding downstream organs resulting in ischemia and organ dysfunction due to the dissection-related aorta and its branch vessel obstruction. Postoperative mortality represented all-cause death within the same hospitalization duration during which the surgery was performed, or after 30 days post-surgery.

### Definitions

The diagnostic criterion for ATAAD was that type A aortic dissection confirmed by CTA within 14 days of onset. ACS include ST-segment elevation myocardial infarction (STEMI), non-ST elevation myocardial infarction (NSTEMI), and unstable angina pectoris (UA). We enrolled mainly NSTEM-ACS patients, including NSTEMI and UA patients, who had typical ischemic chest pain symptoms or dynamic ECG changes, with or without elevated markers of myocardial necrosis. The diagnostic criterion for hypertension was systolic blood pressure ≥140 mmHg and/or diastolic blood pressure ≥90 mmHg. The diagnostic criteria for diabetes mellitus were fasting blood glucose ≥7.0 mmol/L, or randomized blood glucose or glucose load test for 2 h blood glucose ≥11.1 mmol/L, with or without diabetes symptoms: polydipsia, polyuria, weight loss, skin itching, blurred vision and other acute metabolic disorders. Patients' personal disease history and smoking history were based on the admission record and previous diagnosis, including coronary heart disease or coronary interventional therapy history; aortic interventional therapy history; cerebral infarction or hemorrhage history; chronic renal failure or dialysis treatment history.

### Surgical techniques

A median sternum incision was employed for all operations. To conduct aortic reconstruction surgery, the right axillary artery cannulation was performed for cardiopulmonary bypass and selective cerebral perfusion under moderate-to-mild hypothermic circulatory arrest (HCA). First, we carried out proximal aortic root operations, namely, ascending aorta replacement or the Bentall procedure. As stated in our previous studies (21, 22), we employed total arch replacement (TAR) and frozen elephant trunk (FET) to perform extended arch reconstruction. All coronary artery operations utilized the off-pump coronary artery bypass grafting (OPCABG) protocols.

### YAP content evaluation

All evaluations complied with the standard biosecurity and institutional safety procedures. At all blood draws, 2 ml blood was extracted from the peripheral venous tube, then discarded. Subsequently, another 5 ml blood was extracted and collected into a tube containing Ethylene Diamine Tetraacetic Acid, prior to refrigeration at 4°C. Samples then underwent a 20 min centrifugation at 3,000 rpm, and the superstratum serum was transferred to a fresh tube for storage at −80°C. YAP content was assessed using a commercial enzyme-linked immunosorbent assay (ELISA) kit (Human YAP ELISA KIT, Shanghai Fantai Biotechnology Co., Ltd.). Samples were run on TECAN Infinite F50 (TECAN group, Männedorf, Switzerland) and the optical density (OD) values at 450 nm were measured. Lastly, YAP content was calculated according to the calibration curve. YAP contents were evaluated by staff skilled in basic research, who closely followed kit protocols. All examinations were performed in over three repetitions, and the mean of all results were computed for additional analyses.

### Statistical analysis

SPSS 25.0 (IBM Corp., Armonk, NY, USA) was used for all data analyses. Normally distributed continuous data are provided as mean ± standard deviation (SD), non-normally distributed variables as median (interquartile range), and categorical information as numbers (percentage). Differences between cohorts were assessed using the Student's *t*-test, Mann-Whitney *U*-test, Chi-square-test, or Fisher's exac*t*-test. Receiver operating characteristic curves (ROC) were constructed to evaluate the predictive power of bioindicators in early ATAAD diagnosis and in the evaluation of postoperative efficacy. The area under the curve (AUC) quantified the ROC curve. The Youden's index (J = Sensitivity + Specificity-1) was employed to identified the most appropriate cut-off value. All tests were two-sided, with *P* < 0.05 as the significance threshold. Graphs were plotted using GraphPad Prism 8 for Windows (GraphPad software, La Jolla, CA, USA).

## Results

### Patient demographics

Table 1 summarized the baseline profiles of the 60 ATAAD and 50 ACS participants. The mean ATAAD onset age was younger than ACS (48.45 vs. 59.66, *P* < 0.01); and the mean BMI of ATAAD patients was increased, compared to ACS (27.33 vs. 25.76, *P* = 0.03). The most prevalent ATAAD comorbidity was hypertension, occurring in 50 cases (83.33%), which was substantially elevated than in ACS patients (*n* = 32, 64.00%). In contrast, the prevalence of diabetes in ACS patients was 30.00% (*n* = 15), which was strongly elevated than in ATAAD patients (*n* = 3, 5.00%).

**Table 1:** Patient preoperative data.

Variables	ATAAD (*N* = 60)	ACS (*N* = 50)	*P*
Age, years	48.45 ± 10.87	59.66 ± 9.74	<0.01*
Male	43 (71.67)	38 (76.00)	0.61
BMI, kg/m^2^	27.33 ± 4.04	25.76 ± 3.29	0.03*
Hypertension	50 (83.33)	32 (64.00)	0.02*
Diabetes mellitus	3 (5.00)	15 (30.00)	<0.01*
History of coronary artery disease	1 (1.67)		
History of aortic interventional therapy	2 (3.33)	0 (0)	0.50
History of cerebrovascular disease	4 (6.67)	7 (14.00)	0.20
History of chronic renal failure	2 (3.33)	0 (0)	0.50
Smoking history	27 (45.00)	20 (40.00)	0.60
Diameter of the aortic sinus, mm	41.22 ± 6.25	34.44 ± 3.76	<0.01*
BAV	1 (1.67)	0 (0)	1
LVEDD, mm	48.28 ± 6.78	47.56 ± 4.90	0.53
LVEF,%	61.65 ± 5.44	57.12 ± 7.20	<0.01*
AI			<0.01*
No	18 (30.00)	41 (82.00)	<0.05*
Mild	18 (30.00)	8 (16.00)	
Moderate	12 (20.00)	1 (2.00)	<0.05*
Severe	12 (20.00)	0 (0)	<0.05*
Malperfusion			
Cerebrum	12 (21.82)		
Coronary artery	5 (8.77)		
Spinal cord	14 (26.42)		
Abdominal organ	11 (20.75)		
Renal	12 (22.64)		
Lower limbs	6 (11.11)		
Hemopericardium			
No	23 (38.33)		
Yes	29 (48.33)		
Pericardial tamponade	8 (13.33)		
YAP, ng/ml	3.45 (3.18, 3.63)	2.44 (2.23, 2.59)	<0.01*
ALT, U/L	21.00 (15.00, 37.25)	25.50 (17.75, 33.25)	0.71
AST, U/L	22.00 (16.00, 32.75)	19.00 (16.75, 25.75)	0.18
BUN, mmol/L	5.79 (4.70, 7.53)	5.17 (4.51, 6.35)	0.13
Cre, μmol/L	74.60 (62.23, 90.25)	74.30 (61.48, 86.33)	0.57
TNI, pg/ml	10.35 (3.65, 39.73)	10.10 (3.78, 22.85)	0.93
Myo, ng/ml	27.95 (18.38, 50.50)	20.80 (18.40, 30.50)	0.07
WBC, ×10^9^/L	11.46 (9.57, 14.03)	6.24 (5.10, 7.30)	<0.01*
CRP, mg/L	11.62 (3.97, 73.12)	2.87 (0.91, 15.95)	0.03*
Hb, g/L	139.68 ± 19.09	136.41 ± 16.95	0.35
PLT, ×10^9^/L	181.00 (145.50, 214.75)	222.00 (180.50, 275.00)	<0.01*
PT, Sec	11.95 (11.53, 13.05)	11.50 (11.00, 12.10)	<0.01*
APTT, Sec	31.10 (29.03, 34.00)	32.70 (29.90, 34.73)	0.04*
D-Dimer, ng/ml	2,097.00 (998.75, 3,652.00)	97.00 (67.25, 137.00)	<0.01*
Glu, mg/dl	140.20 (118.00, 164.70)	140.50 (120.75, 191.75)	0.42
Lac, mmol/L	1.45 (1.03, 2.45)	1.60 (1.30, 1.93)	0.68

Data are presented as mean ± SD, median [interquartile range] or number (%) as appropriate.

ATAAD, acute type A aortic dissection; ACS, acute coronary syndrome; BMI, body mass index; BAV, bicuspid aortic valve; LVEDD, left ventricular end-diastolic dimension; LVEF, left ventricular ejection fraction; AI, aortic valve insufficiency; YAP, yes-associated protein; ALT, alanine aminotransferase; AST, aspartate aminotransferase; BUN, blood urea nitrogen; Cre, creatinine; TNI, troponin I; Myo, myoglobin; Ams, blood amylase; WBC, white blood cell; CRP, C-reactive protein; Hb, hemoglobin; PLT, platelet; PT, prothrombin time; APTT, activated partial thromboplastin time; Glu, blood glucose value; Lac, lactic acid.

* *P* < 0.05.

### Aortic CTA and ultrasonic cardiogram (UCG)

As detailed in Table 1, relative to the ACS patients, ATAAD patients exhibited a wider aortic sinus diameter (41.22 mm vs. 34.44 mm, *P* < 0.01) and elevated left ventricular ejection fraction (LVEF) (61.65% vs. 57.12%, *P* < 0.01). In case of aortic valve insufficiency (AI), ATAAD patients exhibited substantially more moderate/severe AI (12/60, 20% vs. 1/50, 2%; 12/60, 20% vs. 0/50, 0%, all *P* < 0.05).

### Circulating biomarker contents

**Figure 1:**
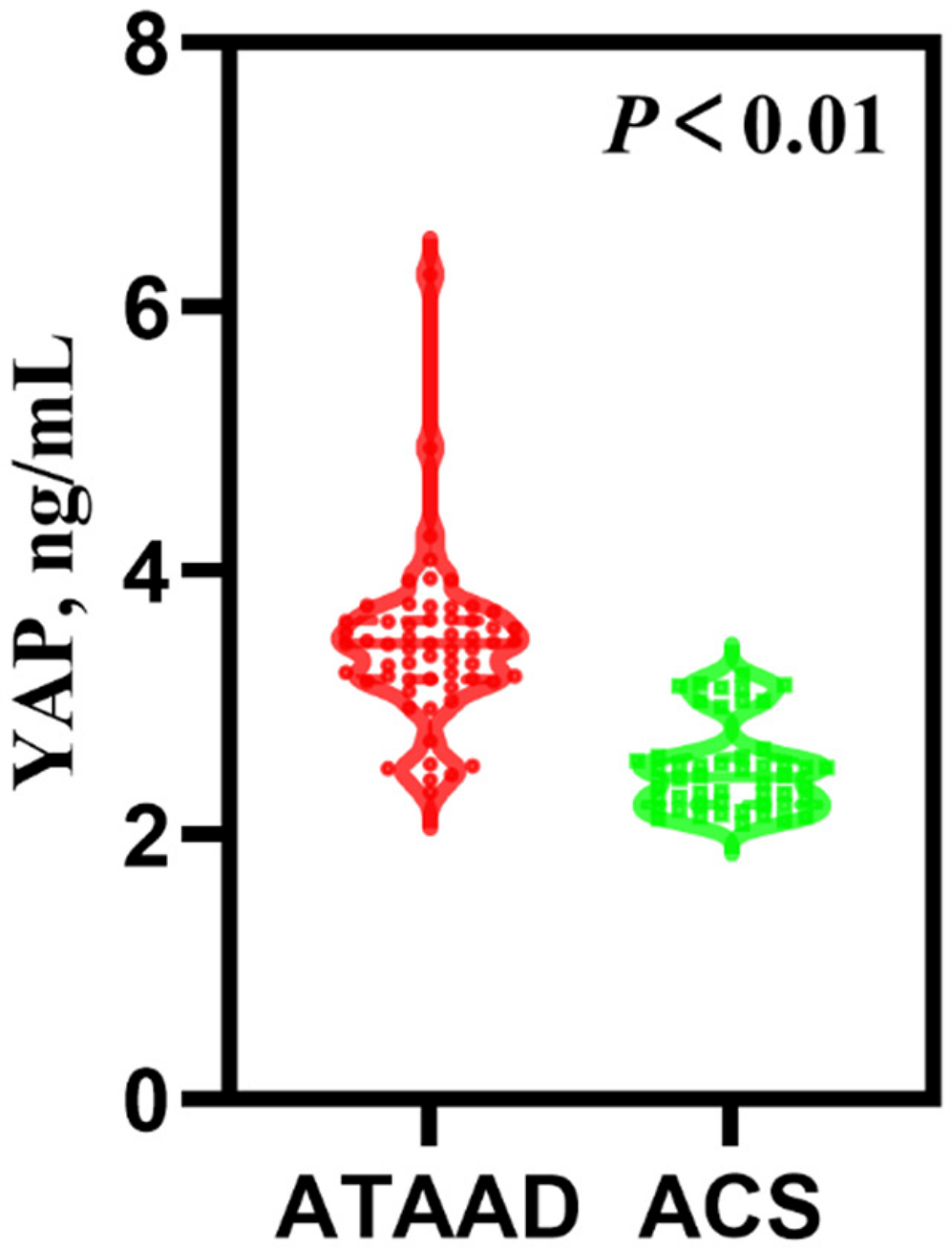
Serum YAP concentrations among ATAAD and ACS patients. [ATAAD: *n* = 60, 3.45 (3.18, 3.63); ACS: *n* = 50, 2.44 (2.23, 2.5); *P* < 0.01].

Table 1 presented the preoperative circulating biomarker concentrations. As depicted in Figure 1, the YAP content in ATAAD patients was remarkably high, compared to the ACS patients [3.45 (3.18, 3.63) vs. 2.44 (2.23, 2.59), *P* < 0.01]. Moreover, the white blood cell (WBC) count and C-reactive protein (CRP) were strongly elevated among ATAAD patients [11.46 (9.57, 14.03) vs. 6.24 (5.10, 7.30), *P* < 0.01; 11.62 (3.97, 73.12) vs. 2.87 (0.91, 15.95), *P* = 0.03]. In terms of coagulation system, the prothrombin time (PT) was enhanced among ATAAD vs. ACS patients [11.95 (11.53, 13.05) vs. 11.50 (11.00, 12.10), *P* < 0.01], the activated partial thromboplastin time (APTT) was diminished among ATAAD vs. ACS patients [31.10 (29.03, 34.00) vs. 32.70 (29.90, 34.73), *P* = 0.04], and the plasma fibrin D-dimers were strongly upregulated among ATAAD vs. ACS patients [2,097.00 (998.75, 3,652.00) vs. 97.00 (67.25, 137.00), *P* < 0.01]. Apart from these, the platelet (PLT) counts were considerably reduced among ATAAD vs. ACS patients [181.00 (145.50, 214.75) vs. 222.00 (180.50, 275.00), *P* < 0.01].

### Logistic regression predictive model

**Figure 2:**
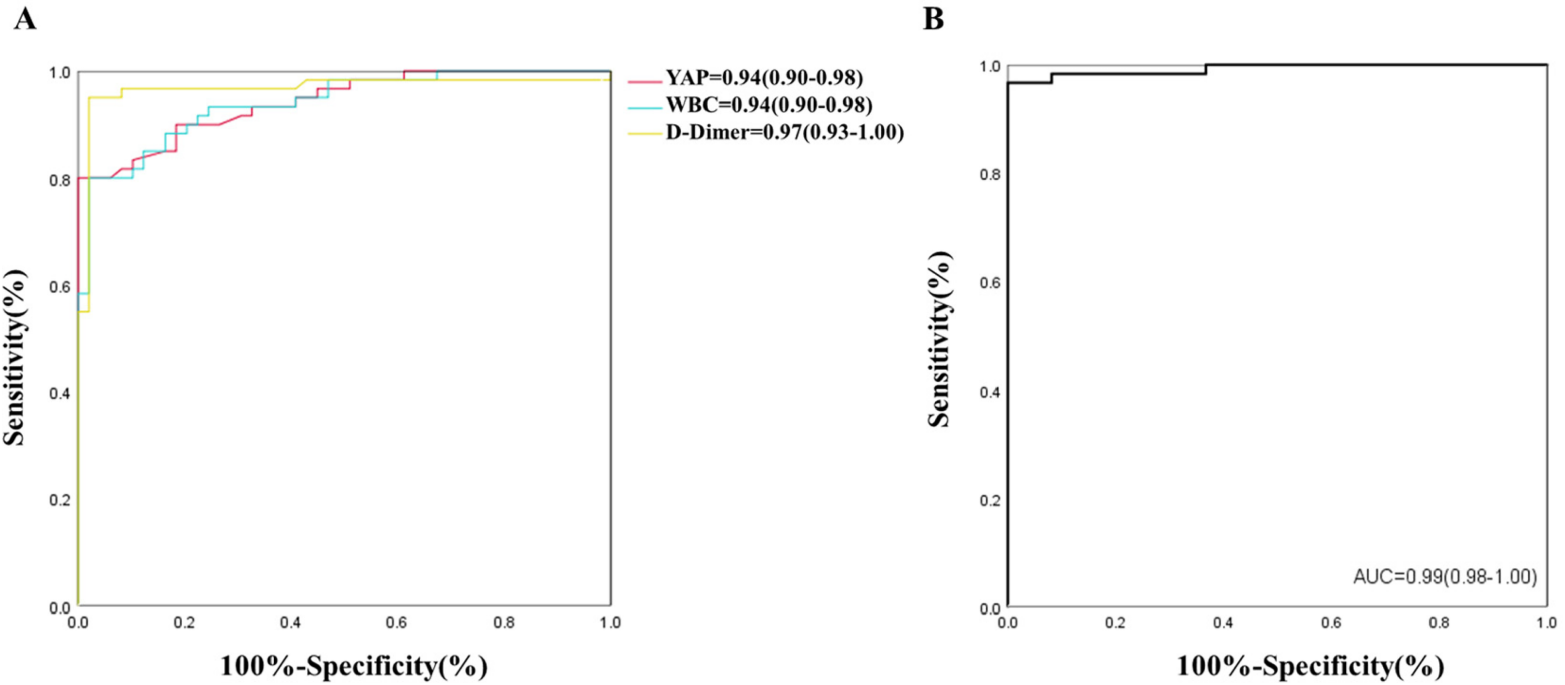
**(A,B)** ROC of YAP, WBC, D-Dimer and multiple indexes for detection of ATAAD.

Following statistical evaluation, all logistics regression equation variables satisfied tolerance >0.1 and variance expansion coefficient <10. Therefore, there is no multicollinearity between all variables. Using the forward: LR independent variable filtering method, we next integrated the YAP, WBC count and D-Dimer into the logistic regression predictive model. Furtherly, ROC analysis was performed for single and multiple measurement indexes, and the biomarkers cutoff values, sensitivities, specificities and predictive values were measured. As depicted in Figure 2A and Table 2, ROC analysis yielded the optimal cutoff value was 3.15 ng/ml for YAP, with an AUC of 0.94 (95% CI, 0.90–0.98, *P* < 0.01), a sensitivity of 80% and a specificity of 98% for ATAAD identification.

**Table 2:** ROC analysis results of YAP, WBC, D-Dimer for detection of ATAAD.

Variables	Cut-off value	Sensitivity	Specificity	AUC	95% CI of AUC	*P*
YAP, ng/ml	3.15	0.80	0.98	0.94	0.90–0.98	<0.01
WBC, ×10^9^/L	9.33	0.80	0.98	0.94	0.90–0.98	<0.01
D-Dimer, ng/ml	392.50	0.95	0.98	0.97	0.93–1.00	<0.01

YAP, yes-associated protein; WBC, white blood cell; ATAAD, acute type A aortic dissection.

In case of WBC count and D-Dimer, the ROC analysis estimated the optimal cutoff value of 9.33 × 10^9^/L for WBC count, with a sensitivity and specificity of 80% and 98%, respectively; and the optimal cutoff value of 392.50 ug/L for D-dimer, with a sensitivity and specificity of 95% and 98%, respectively. The ROC analysis model combining with all three measurement indexes (YAP, WBC count and D-Dimer) was shown in Figure 2B, the predictive power was significantly improved, with an AUC of 0.99 (95% CI, 0.98–1.00, *P* < 0.01).

### Pre- and intraoperative ATAAD patient data

Table 3 listed the information of 60 ATAAD patients. Compared to the non-MAEs counterparts, MAEs patients presented with remarkably augmented levels of blood urea nitrogen (BUN) [5.56 (4.63, 6.48) vs. 7.50 (6.06, 10.86), *P* < 0.01], creatinine (Cre) [72.90 (59.85, 85.45) vs. 125.70 (69.00, 137.40), *P* = 0.01], troponin I (TNI) [8.95 (3.13, 31.63) vs. 37.10 (17.90, 97.70), *P* = 0.01], myoglobin (Myo) [24.15 (17.65, 38.93) vs. 74.60 (32.95, 201.28), *P* < 0.01], and blood amylase (Ams) [41.60 (28.00, 58.10) vs. 65.80 (46.13, 74.95), *P* = 0.02]. Intraoperatively, MAEs patients experienced shorter antegrade cerebral perfusion (ACP) duration [30.00 (20.00, 36.00) vs. 19.50 (17.25, 23.25), *P* = 0.04]. Apart from the aforementioned parameters, there were no more marked differences between the two patient cohorts.

**Table 3:** Pre- and intraoperative characteristics of ATAAD patients.

Variables	Non-MAEs (*N* = 49)	MAEs (*N* = 11)	*P*
Age, years	48.10 ± 10.23	50.00 ± 13.83	0.61
Male	37 (75.51)	6 (54.55)	0.31
BMI, kg/m^2^	27.48 ± 4.04	26.65 ± 4.19	0.54
Hypertension	42 (85.71)	8 (72.73)	0.55
Diabetes mellitus	2 (4.08)	1 (9.09)	0.46
Coronary artery disease	1 (2.04)	0 (0)	1
History of aortic interventional therapy	2 (4.08)	0 (0)	1
History of cerebrovascular disease	3 (6.12)	1 (9.09)	0.57
Nephrosis	1 (2.04)	1 (9.09)	0.34
Smoking history	21 (42.86)	6 (54.55)	0.71
Diameter of the aortic sinus, mm	40.00[37.00, 45.00)	39.00[35.00, 43.00)	0.44
BAV	1 (2.04)	0 (0)	1
LVEDD, mm	48.61 ± 5.81	46.82 ± 10.30	0.59
LVEF,%	61.73 ± 5.39	61.27 ± 5.93	0.80
AI			0.38
No	17 (34.69)	1 (9.09)	
Mild	14 (28.57)	4 (36.36)	
Moderate	9 (18.37)	3 (27.27)	
Severe	9 (18.37)	3 (27.27)	
Malperfusion			
Cerebrum	8 (17.02)	4 (50.00)	0.10
Coronary artery	4 (8.51)	1 (9.09)	1
Spinal cord	11 (23.40)	3 (50.00)	0.32
Abdominal organ	8 (17.02)	3 (50.00)	0.10
Renal	9 (19.15)	3 (50.00)	0.12
Lower limbs	5 (10.64)	1 (14.29)	1
Hemopericardium			0.65
No	18 (36.73)	5 (45.45)	
Yes	25 (51.02)	4 (36.36)	
Pericardial tamponade	6 (12.24)	2 (18.18)	
ALT, U/L	19.00 (15.00, 36.50)	31.00 (20.00, 38.00)	0.49
AST, U/L	22.00 (16.00, 27.50)	45.00 (16.00, 60.00)	0.20
BUN, mmol/L	5.56 (4.63, 6.48)	7.50 (6.06, 10.86)	<0.01*
Cre, μmol/L	72.90 (59.85, 85.45)	125.70 (69.00, 137.40)	0.01*
TNI, pg/ml	8.95 (3.13, 31.63)	37.10 (17.90, 97.70)	0.01*
Myo, ng/ml	24.15 (17.65, 38.93)	74.60 (32.95, 201.28)	<0.01*
Ams, U/L	41.60 (28.00, 58.10)	65.80 (46.13, 74.95)	0.02*
Glu, mg/dl	140.40 (118.00, 162.40)	138.60 (117.00, 203.40)	0.61
Lac, mmol/L	1.50 (1.00, 2.40)	1.40 (1.20, 4.30)	0.50
Operation duration, h	6.58 ± 1.18	6.53 ± 1.34	0.91
CPB duration, min	174.00 (155.50, 203.00)	187.00 (163.25, 238.25)	0.19
Cross-clamp duration, min	96.67 ± 24.03	96.50 ± 42.46	0.99
Nasopharyngeal temperature, °C	25.20 (24.65, 29.50)	25.70 (25.00, 28.20)	0.65
Bladder temperature, °C	27.80 (26.35, 31.00)	27.70 (25.78, 30.53)	0.70
Perfusion pressure during ACP, mmHg	60.00 (60.00, 70.00)	60.00 (60.00, 72.50)	0.93
ACP flow, ml/(kg·min)	6.67 (5.25, 8.57)	7.62 (5.00, 8.57)	0.83
Circulatory arrest duration, min	23.00 (16.00, 33.00)	19.00 (14.50, 23.25)	0.27
ACP duration, min	30.00 (20.00, 36.00)	19.50 (17.25, 23.25)	0.04*

Data are presented as mean ± SD, median [interquartile range], or number (%) as appropriate.

ATAAD, acute type A aortic dissection; MAEs, major adverse events; BMI, body mass index; BAV, bicuspid aortic valve; LVEDD, left ventricular end-diastolic dimension; LVEF, left ventricular ejection fraction; AI, aortic valve insufficiency; ALT, alanine aminotransferase; AST, aspartate aminotransferase; BUN, blood urea nitrogen; Cre, creatinine; TNI, troponin I; Myo, myoglobin; Ams, blood amylase; Glu, blood glucose value; Lac, lactic acid; CPB, cardiopulmonary bypass; ACP, antegrade cerebral perfusion.

* *P* < 0.05.

### Postoperative mortality and morbidity

Table 4 detailed the postoperative outcomes of ATAAD patients. MAEs patients took considerably longer to achieve consciousness [3.33 (2.00, 5.21) vs. 16.09 (4.21, 38.56), *P* < 0.01], and they had prolonged ventilation duration [13.50 (9.67, 20.30) vs. 47.20 (14.12, 101.00), *P* = 0.02], relative to non-MAEs patients. Moreover, the postoperative AKI with CRRT and mortality incidences were considerably elevated in MAEs vs. non-MAEs patients.

**Table 4:** Postoperative mortality and morbidity.

Variables	Non-MAEs (*N* = 49)	MAEs (*N* = 11)	*P*
In-hospital time, days	13.00 (10.50, 16.00)	10.00 (6.00, 21.00)	0.36
Consciousness recovery time, h	3.33 (2.00, 5.21)	16.09 (4.21, 38.56)	<0.01*
Ventilation time, h	13.50 (9.67, 20.30)	47.20 (14.12, 101.00)	0.02*
ICU stay time, h	42.42 (19.33, 67.54)	35.58 (23.92, 272.75)	0.45
TND	13 (26.53)	5 (45.45)	0.38
PND	0 (0)	1 (9.09)	0.18
AKI with CRRT	0 (0)	6 (54.55)	<0.01*
Re-intubate the ventilator	0 (0)	1 (9.09)	0.18
Tracheotomy	0 (0)	1 (9.09)	0.18
Paraplegia	0 (0)	1 (9.09)	0.18
Monoplegia of lower limbs	0 (0)	1 (9.09)	0.18
Mortality	0(0)	4(36.36)	<0.01*

Data are presented as median [interquartile range], or number (%) as appropriate.

MAEs, major adverse events; ICU, intensive care unit; TND, temporary neurological dysfunction; PND, permanent neurological dysfunction; AKI, acute kidney injury; CRRT, continuous renal replacement therapy.

* *P* < 0.05.

### Association between Serum YAP values and postoperative MAEs

**Figure 3:**
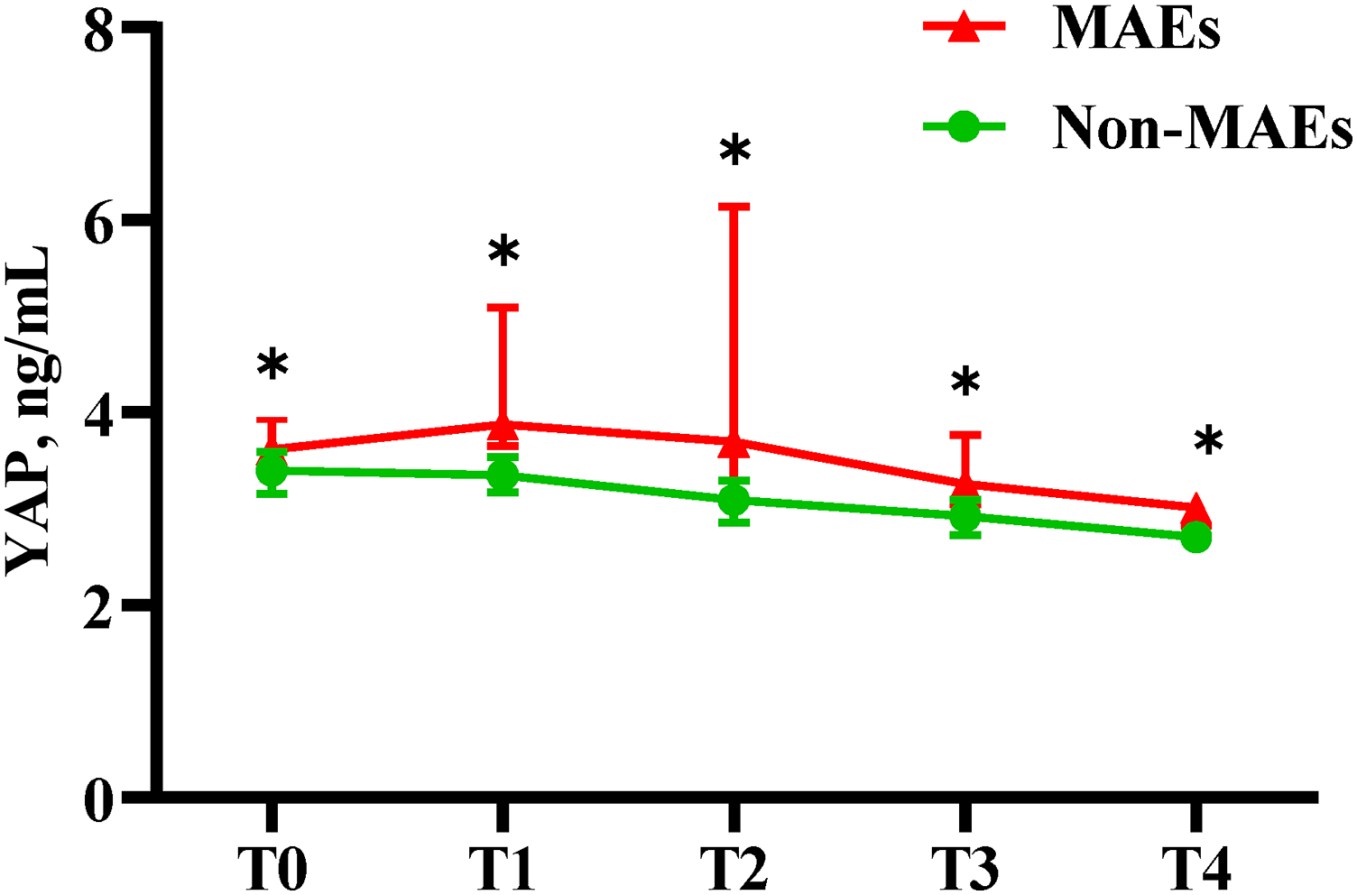
Serum YAP values of ATAAD patients at each study time point. Non-parametric test: *P* < 0.05(*).

**Figure 4:**
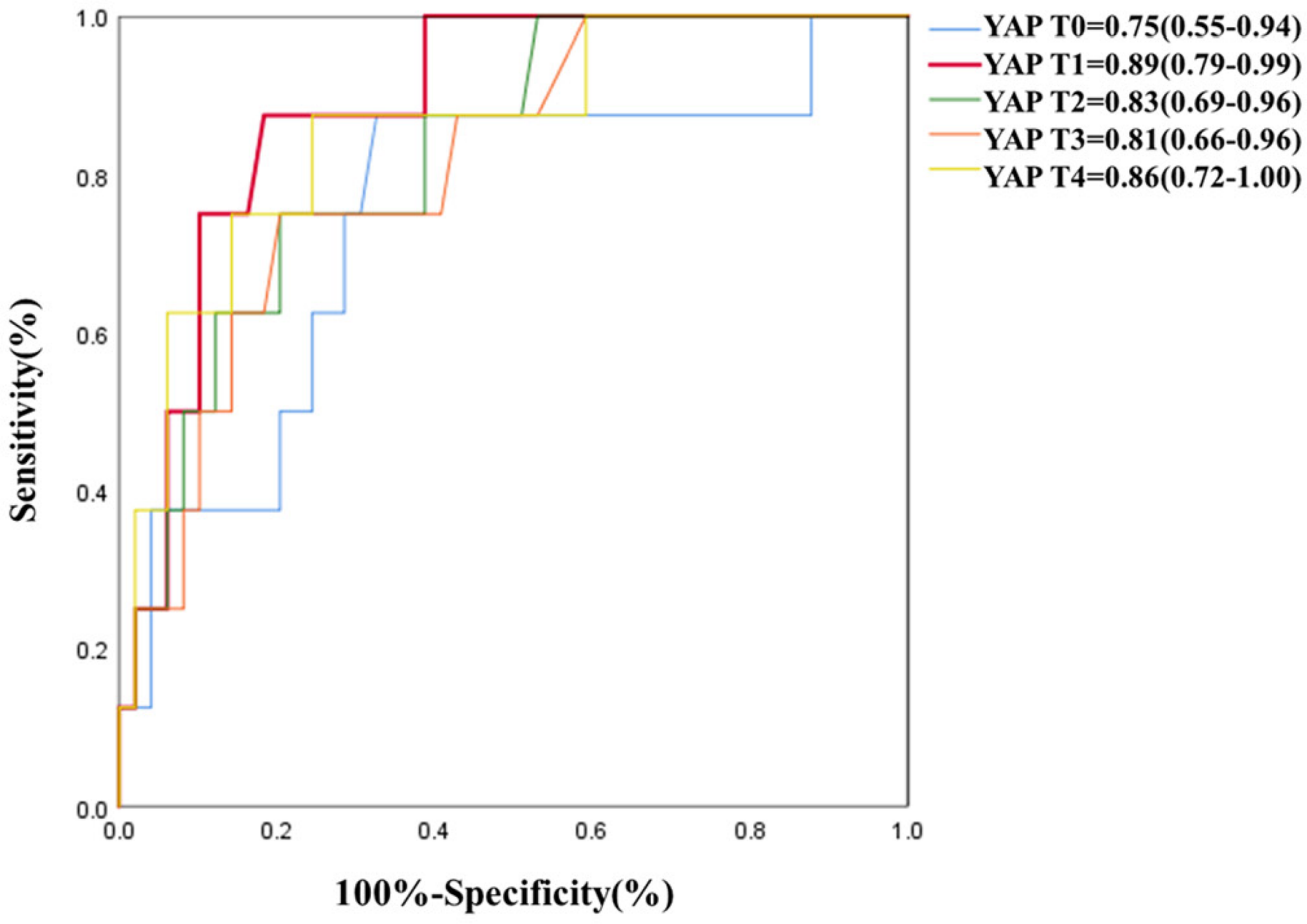
ROC of YAP at each study time point for predictive diagnosis of MAEs.

As depicted in Table 5 and Figure 3, serum YAP levels among MAEs patients were substantially elevated, compared to non-MAEs patients at all examined time points (all *P* < 0.01). Therefore, we next conducted ROC analyses on YAP values at each pre-specified time point, to assess the predictive power of YAP in early postoperative outcomes of ATAAD patients. Based on the results in Figure 4 and Table 6, the YAP predictability reached the highest peak at 12 h (T1) post-surgery, with an AUC of 0.89 (95% CI, 0.79–0.99, *P* < 0.01), and sensitivity and specificity of 88% and 82%, respectively. The optimal cutoff value of YAP was 3.60 ng/ml at predicting MAEs at 12 h post-surgery. Based on the aforementioned evidences, YAP content is a robust risk predictor for postoperative MAEs among ATAAD patients.

**Table 5:** Serum YAP values of ATAAD patients at each study time point.

Variables	Non-MAEs (*N* = 49)	MAEs (*N* = 11)	*P*
YAP T0, ng/ml	3.40 (3.16, 3.59)	3.62 (3.49, 3.94)	<0.01*
YAP T1, ng/ml	3.35 (3.17, 3.54)	3.88 (3.66, 5.09)	<0.01*
YAP T2, ng/ml	3.09 (2.86, 3.30)	3.70 (3.30, 6.14)	<0.01*
YAP T3, ng/ml	2.93 (2.73, 3.10)	3.26 (3.05, 3.77)	<0.01*
YAP T4, ng/ml	2.71 (2.58, 2.81)	3.01 (2.83, 3.12)	<0.01*

Data are presented as median [interquartile range].

YAP, yes-associated protein; ATAAD, acute type A aortic dissection.

* *P* < 0.05.

**Table 6:** ROC analysis result of YAP at 12 h (T1) post-surgery for predictive diagnosis of MAEs.

Variables	Cut-off value	Sensitivity	Specificity	AUC	95% CI of AUC	*P*
YAP T1, ng/ml	3.60	0.88	0.82	0.89	0.79–0.99	<0.01

YAP, yes-associated protein; MAEs, major adverse events.

## Discussion

Herein, we evaluated circulating YAP contents in ATAAD and ACS patients, and examined the predictive power of YAP in detecting AAD and predicting postoperative MAEs. Our analysis uncovered the following: (1) Serum YAP level is strongly upregulated among ATAAD vs. ACS patients; (2) Serum YAP level is a potent biomarker for early AAD diagnosis; (3) Serum YAP level is a strong indicator of postoperative MAEs after aortic reconstruction surgery. Given these evidences, the circulating YAP concentration following sudden-onset severe chest pain, in combination with the WBC count and D-Dimer levels, is a potentially robust tool for detecting AAD, which can ultimately enhance AAD diagnosis and timely intervention. In addition, we revealed that YAP concentration at 12 h post-surgery was a strong indicator of early postoperative outcomes, which can greatly enhance short-term complication management and improve patient prognosis.

YAP is a transcriptional coregulator that serves as the key and major effectors of the Hippo pathway, and the upstream kinase cascades also include the MST1/2 (mammalian Ste20-like kinases 1/2) and LATS1/2 (large tumor suppressor 1/2) (23). Under the Hippo way inactive conditions, YAP is unphosphorylated and translocated into the nucleus, whereby it interacts with the TEAD (transcriptional enhanced associate domain) transcription factors to promote expression of a number of genes related to cellular proliferation, survival, and migration (24–26). Upon the Hippo way activation, MST1/2-activated LATS1/2 phosphorylates YAP, which then accumulates in the cytoplasm, where it is eventually degraded (27).

YAP is an important mechanoregulatory transcriptional effector *in vivo*, relaying physical cues to gene expression and cellular responses. In the cardiovascular system, YAP has been reported to mediate inflammatory responses and vascular remodeling. Different patterns of blood flow could have different effects on the activities of YAP. It was previously revealed that laminar shear stress inactivates YAP, whereas proliferative/proinflammatory oscillatory shear stress maintains YAP activities (28, 29). Enhanced arterial wall stress or biomechanical stress induces SMCs phenotypic switching during vascular remodeling via activation of the YAP signaling pathway (30). On the onset of AAD, the mutation of blood flow pattern impacts both YAP activity and signaling. Zhang C et al. (31) also reported that aortic biomechanical stress triggers the systemic epigenetic induction of an adaptive response (e.g., wound healing, proliferation, matrix organization) in aortic SMCs, and YAP was identified as a key TF (transcription factor) driving this adaptive response.

In a previous investigation, we demonstrated that ATAAD aortic wall samples exhibit marked ECM disorders, enhance VSMCs apoptosis, and strongly diminish YAP content. Moreover, the YAP downregulation is induced by mechanical stress disruption (17). Xie C et al. (32) reported that the YAP signaling pathway is a central regulator of phenotypic switch of VSMCs. Wang X et al. (33) also demonstrated that YAP is crucial for smooth muscle phenotypic modulation and neointima formation by promoting VSMCs proliferation and migration after arterial injury. Likewise, in a study conducted by Liu T et al. (34), YAP played a critical role in affecting VSMCs apoptosis and AD pathogenesis, while up-regulating YAP to suppress AD formation via suppression of VSMC apoptosis. AD down-regulates YAP by activating the Hippo pathway and increasing YAP phosphorylation in VSMCs (35). Together, these findings provide strong evidence that the Hippo-YAP pathway could be a potential key pathophysiological mediator of dissection.

The key role of YAP in AD pathogenesis has been extensively studied. Nevertheless, there are limited reports on the influence of circulating YAP distribution on the acute onset and perioperative periods of ATAAD patients, thus, extensive research is still warranted on these areas of research. Herein, during the acute periods, the median circulating YAP content among ATAAD patients was almost 1.5 times higher than ACS patients (3.45 vs. 2.44 ng/ml). We speculated that this rise in circulating YAP levels was likely due to the aortic wall tissue injury and associated inflammatory responses following AAD. Both mechanically damaged and compromised ECM can significantly alter resident cell phenotype or enhance infiltration of inflammatory cells (36, 37). At the onset of aortic dissection, blood flow immediately rushes into the false lumen through the intimal rupture. Owing to alterations within the aortic hemodynamics, this event increases aortic media exposure and destruction. VSMCs face direct exposure to blood flow, shear stress and local inflammation (38). Both the integrin α5β1/c-Abl pathway and the integrin/Gα13/RhoA/LATS1/2 pathway are involved in the proinflammatory disturbed flow upregulation of YAP (28, 29). Among dissection patients, VSMCs stretch and vascular injury occurred in large area of aorta, the largest artery, which, may, in turn, enhance YAP accumulation in the circulation better than other intravascular damage, such as ACS or APE.

As for other serum markers: D-dimer, which has been routinely considered as an exclusionary biomarker for the acute phase of AAD (7–9); WBC count is one of the classic blood routine abnormal biomarkers of acute systemic severe inflammation outbreak in all critical emergency patients (37, 39). Both of these values are abnormally elevated in the acute phase of AAD, but there are inevitable limitations in the accuracy of early diagnosis. The increase of D-dimer is dependent on the over-activation of the fibrinolytic system and, therefore, is also common in other thrombotic or clotting disorders such as APE (10, 11). The high level of WBC count is due to a severe systemic inflammatory response, and obviously, it is also not a specific biomarker. The increase of circulating YAP levels can reflect the change of arterial wall stress and blood flow pattern caused by AAD, and further improve the specificity of AAD diagnosis. Therefore, YAP plays a key and indispensable role in this diagnostic system, which is worthy of further in-depth study.

Patient prognosis following AAD repair surgery depends heavily on the restoration of blood supply and organ functions throughout the body. Potential postoperative organ malperfusion or ischemic necrosis is challenging to detect early using imaging examination. A rise in circulating YAP levels among patients with postoperative MAEs may be linked to postoperative systemic inflammatory response or ischemic injury of organs. Ischemia, hypoxia injury and cellular necrosis of various organs following surgery can further enhance the release of inflammatory factors (39, 40). In this study, we observed that the median circulating YAP contents among patients with MAEs at 12 h post-surgery was considerably elevated, compared to before surgery. Additionally, relative to non-MAEs patients, the postoperative serum YAP decline was slower in MAEs patients. This distinct serum YAP profile may indicate enhanced inflammatory or ischemic injury following surgery. The serum YAP levels at 12 h post-surgery also provided the most accurate prediction of postoperative MAEs in ATAAD patients.

Based on our literature screening, this study is the first to report on the peripheral blood distribution profile of the biomarker YAP among ATAAD patients during the acute phase. Our preliminary results indicated that YAP is a potentially robust early bioindicator of AAD, owing to its successful discrimination AAD from ACS following symptom onset. Thus, serum YAP concentration can potentially be used to make early diagnosis of patients with acute chest pain and suspected AAD. Furthermore, postoperative serum YAP content can also aid in the early prediction of postoperative MAEs. However, the YAP significance in peripheral blood pathologies following aortic dissection remains to be evaluated in future investigations.

This research has certain limitations. Firstly, it was conducted in a single center, using a relatively small population size. This report assessed various circulating biomarkers in well-defined patient groups. However, we are unable to completely eliminate influence of selection bias from our results. Hence, additional explorations are warranted involving a larger prospective multicenter cohort study to validate the accuracy of our results. Moreover, prior to clinical application, it is imperative to confirm the threshold values of serum YAP concentration to establish reliable laboratory reference ranges for the early diagnosis and postoperative MAEs prediction of AAD patients.

## Conclusion

Herein, we demonstrated that serum YAP level is a robust early biomarker for AAD diagnosis, that it clearly delineates AAD from ACS at the onset of chest pain. Moreover, the circulating YAP levels can also be used to predict postoperative MAEs. Our findings require further validation by a large-scale prospective clinical trial prior to its usage in a clinical setting.

## Data Availability

The raw data supporting the conclusions of this article will be made available by the authors, without undue reservation.
